# Sociodemographic Differences in Logins and Engagement With the Electronic Health Coach Messaging Feature of a Mobile App to Support Opioid and Stimulant Use Recovery: Results From a 1-Month Observational Study

**DOI:** 10.2196/54753

**Published:** 2025-04-10

**Authors:** Lindsey M Filiatreau, Hannah Szlyk, Alex T Ramsey, Erin Kasson, Xiao Li, Zhuoran Zhang, Patricia Cavazos-Rehg

**Affiliations:** 1 Division of Infectious Diseases School of Medicine Washington University in St Louis St Louis, MO United States; 2 Department of Psychiatry School of Medicine Washington University in St Louis St Louis, MO United States

**Keywords:** substance misuse, substance use recovery, opioid use disorder, stimulant use disorder, uptake, engagement, mHealth, digital health intervention, sociodemographic, mobile app, stimulant use, observational study, mobile health, smartphone, St. Louis, eCoach messaging, Wilcoxon rank-sum tests, Pearson chi-square, recovery, app

## Abstract

**Background:**

Mobile health apps can serve as a critical tool in supporting the overall health of uninsured and underinsured individuals and groups who have been historically marginalized by the medical community and may be hesitant to seek health care. However, data on uptake and engagement with specific app features (eg, in-app messaging) are often lacking, limiting our ability to understand nuanced patterns of app use.

**Objective:**

This study aims to characterize sociodemographic differences in uptake and engagement with a smartphone app (uMAT-R) to support recovery efforts in a sample of individuals with opioid and stimulant use disorders in the Greater St. Louis area.

**Methods:**

We enrolled individuals into the uMAT-R service program from facilities providing recovery support in the Greater St. Louis area between January 2020 and April 2022. Study participants were recruited from service project enrollees. We describe the number of logins and electronic health coach (eCoach) messages participants sent in the first 30 days following enrollment using medians and IQRs and counts and proportions of those who ever (vs never) logged in and sent their eCoach a message. We compare estimates across sociodemographic subgroups, by insurance status, and for those who did and did not participate in the research component of the project using Wilcoxon rank-sum tests and Pearson chi-square tests.

**Results:**

Of all 695 participants, 446 (64.2%) logged into uMAT-R at least once during the 30 days following enrollment (median 2, IQR 0-8 logins). Approximately half of those who logged in (227/446) used the eCoach messaging feature (median 1, IQR 0-3 messages). Research participants (n=498), who could receive incentives for app engagement, were more likely to log in and use the eCoach messaging feature compared to others (n=197). Younger individuals, those with higher educational attainment, and White, non-Hispanic individuals were more likely to log in at least once compared to their counterparts. The median number of logins was higher among women, and those who were younger, employed, and not on Medicaid compared to their counterparts. Among those who logged in at least once, younger individuals and those with lower educational attainment were more likely to send at least one eCoach message compared to others.

**Conclusions:**

Mobile apps are a viable tool for supporting individuals in recovery from opioid and stimulant use disorders. However, older individuals, racial and ethnic minorities, and those with lower educational attainment may need additional login support, or benefit from alternative mechanisms of recovery support. In addition, apps may need to be tailored to achieve sustained engagement (ie, repeat logins) among men, and individuals who are older, unemployed, or on Medicaid. Older individuals and those with higher educational attainment who may be less likely to use eCoach messaging features could benefit from features tailored to their preferences.

## Introduction

Unmet health care needs in the United States have increased over the past 2 decades despite overall increases in insurance coverage [1]. The most recent national estimates (2017) suggest the proportion of individuals who were unable to see a physician because of cost was over 15% in the population overall and over 30% among uninsured individuals [1]. In the last 15 years, mobile health (mHealth) technologies have been leveraged to address this issue and support improved physical and mental well-being (eg, weight loss, diabetes management, depression symptom management, substance misuse recovery) nationally [2-5]. mHealth apps are often heralded as a critical tool in supporting the mental and physical health of un- and underinsured individuals and groups, such as racial, ethnic, gender, and sexual minorities who have been historically marginalized by the medical community and may be hesitant to seek care from formal care systems [6-10].

Results from numerous randomized controlled trials have demonstrated the efficacy of mHealth tools in improving a range of health outcomes, including weight loss [11], stress reduction [12], smoking cessation [13], and recovery from substance use disorders (SUDs) [4,14]. For example, a systematic review of digital interventions to support people in recovery from SUDs demonstrated that 70%-90% of individuals found these interventions to be useful and easy to use, and more than half of the studies with control groups demonstrated efficacy in improving recovery-related outcomes [4]. Often, mHealth tools integrate a host of features to allow users to engage in diverse activities such as identifying local community-based resources to support their health; goal and reminder setting; and symptom monitoring, each of which is designed to support healthy behaviors [5,15]. Yet, existing findings suggest uptake and engagement with mHealth apps can vary dramatically [5,15]. Moreover, data on the efficacy, acceptability, and utilization of distinct app features is often lacking, thereby limiting our ability to understand what works for whom, when, and under what settings; importantly, this also limits our understanding of which groups of individuals are missed by unique mHealth strategies [16-18]. As we continue to refine mHealth tools to support overall public health and well-being, understanding the profiles of app users, including users of distinct app features such as in-app messaging, can help fill an important gap in the literature.

The national opioid epidemic has reached new heights with over 105,000 overdose deaths reported in 2021– 80,000 of which were opioid-related [19]. To address this continued and increasing public health crisis, the Substance Abuse and Mental Health Services Administration (SAMHSA) developed the State Targeted Response to the Opioid Crisis Grant in 2017, which funded 57 states and territories to support the provision of prevention, treatment, and recovery programs for people with opioid use disorders (OUDs) [20]. In Missouri, these funds were used to increase access to medication-assisted therapy for OUD, provider-focused overdose prevention initiatives, recovery support services for people with OUD, and to improve the sustainability of such programs through policy implementation [20]. One such recovery support tool funded through the Missouri Opioid State Targeted Response grant is a smartphone app, coined uMAT-R, that was designed to support individuals in recovery from OUDs and stimulant use disorders.

In this study, we aimed to characterize uptake of and overall engagement with the uMAT-R app and in-app electronic health coach (eCoach) messaging feature among those linked to services through the Missouri Opioid State Targeted Response grant funding. We also explore sociodemographic differences in uptake and engagement patterns in distinct subgroups of users to elucidate what types of interventions or adaptations might be needed to improve use in distinct groups of individuals.

## Methods

### Study Procedures

Recruitment for the uMAT-R mobile app intervention program was conducted by study staff in partnership with facilities providing recovery support services for individuals with OUD and other SUDs (eg, SUD treatment programs, Federally Qualified Health Centers) and through the justice system between January 1, 2020, and April 1, 2022. While uMAT-R program staff aimed to foster positive collaborations with recovery facility staff so these individuals would encourage participation in uMAT-R, participation in uMAT-R was voluntary and separate from facility-based recovery services. To be eligible for inclusion in the uMAT-R intervention program, individuals had to: (1) report a history of or current opioid or stimulant misuse; (2) be in treatment at one of the included recruitment facilities; (3) be 18 years or older; (4) be a US resident; (5) be fluent in English; and (6) own or have access to a smartphone with an iOS or Android operating system. Those enrolled in the intervention program were also invited to participate in a 1-month study to ascertain the effect of uMAT-R engagement on recovery outcomes. All individuals enrolled in the service program were provided access to uMAT-R regardless of participation in the research component of the program. Upon enrollment, individuals were provided a unique username and link to access uMAT-R by study staff.

### uMAT-R Intervention

**Figure 1:**
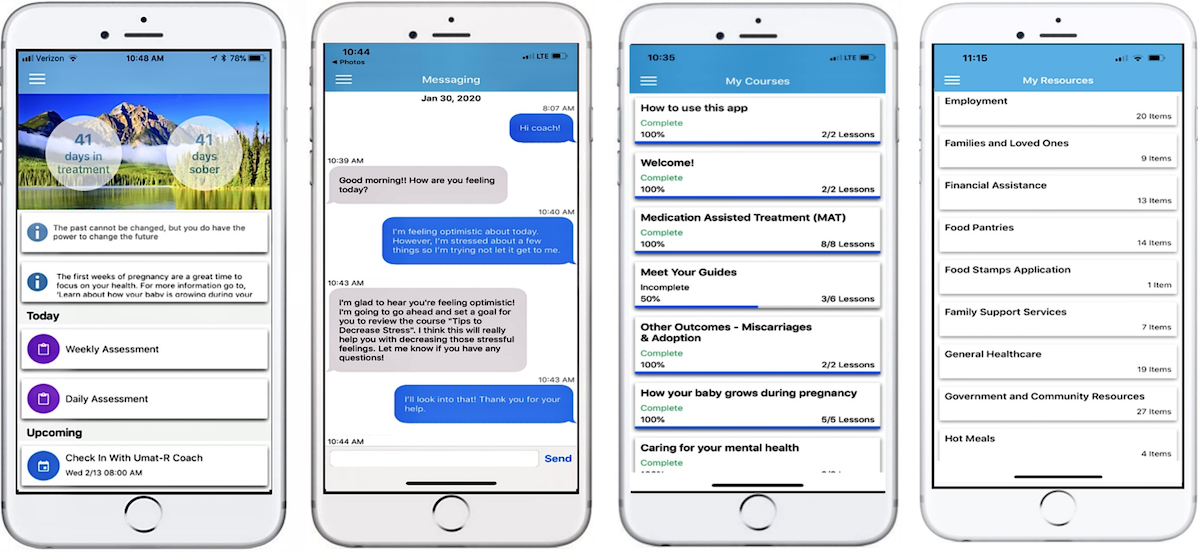
Example images of the uMAT-R app (home page, messaging feature, educational library, resource directory).

The uMAT-R intervention app was developed based on content from the handbook “Decisions in Recovery: Treatment for Opioid Use Disorders” created by SAMHSA, as previously described [21]. This handbook provides information on OUD treatment options, including information regarding costs and potential side effects of distinct treatments (ie, buprenorphine, methadone, and naltrexone) [22]. The app is comprised of several unique features (Figure 1) including over 40 distinct educational courses on topics ranging from “What is Opioid Use Disorder” to “Boundary Setting and Medication for Addiction Treatment” and an extensive directory of community resources throughout Missouri. Additional uMAT-R features to support recovery include goal setting; appointment and medication reminders; sober day tracking; daily motivational quotes; and an in-app messaging feature to communicate with a personal eCoach who is a trained member of the project team and provides near real-time support focused on recovery or essential needs (eg, housing, health care, transportation). The eCoach is also responsible for helping participants navigate any difficulties accessing the app or specific app features.

### Survey Data

Participants providing verbal or written consent were sent a unique link to an electronic copy of a baseline survey which included questions on sociodemographic background, substance use history, previous recovery efforts, and mental health symptoms. All surveys were self-administered and completed in the Research Electronic Data Capture (REDCap, Vanderbilt University) system, a secure, web-based application.

### Uptake and Engagement Data

Uptake and engagement data were ascertained for all individuals assigned a uMAT-R username regardless of participation in the research component of the program. Data included information on the number and time each user logged into the app and the date and time of messages exchanged with an eCoach through the app. We characterized uptake by creating a dichotomous variable to represent any vs no logins. We characterized engagement using three separate measures, a dichotomous variable representing any vs no eCoach messages sent by participants, and two continuous measures representing the total number of logins and eCoach messages sent. Uptake and engagement data were obtained from iTether, the technology company supporting the design and maintenance of the uMAT-R app during the observation period.

### Sociodemographic Characteristics

We used self-reported survey data on gender (men vs women), age (<50 vs ≥50 years), race and ethnicity (non-Hispanic White people vs non-White or Hispanic people), educational attainment (<high school diploma vs high school diploma, General Education Development—a high school equivalency exam in the United States, or greater), employment status (unemployed vs employed part- or full-time), housing status (stably housed [living in own or someone else’s home or apartment] vs unstably housed [homeless or living in a treatment facility]), and insurance status (private insurance vs no private insurance; Medicaid vs no Medicaid; and insurance coverage vs no insurance coverage) among those who chose to engage in the research component of this service project.

### Analyses

Eligible individuals were excluded if their uptake and engagement data were unavailable. Among included individuals, basic uptake and engagement activity was characterized using login and eCoach message data in the 30-day period following enrollment into the service project. Counts and proportions were used to describe app uptake and those who ever versus never used the eCoach messaging feature. Other engagement outcomes related to the total number of logins and eCoach messages sent by participants were characterized using medians and IQRs. Data on the number of eCoach messages sent by participants were restricted to include individuals who logged into uMAT-R at least once.

We then linked uptake and engagement data with baseline participant survey data for those who enrolled in the research component of the project and completed a baseline survey within 7 days of enrollment (to ensure measurement of exposures of interest preceded outcomes of interest) using unique user IDs assigned by iTether. We explored differences in uptake and engagement among those who did and did not consent to participate in the research component of the program and, among those who completed a baseline survey, differences in uptake and engagement in distinct sociodemographic subgroups. We compared estimates of uptake and engagement for distinct sociodemographic subgroups and the group of individuals who did and did not participate in the research component of the program using Wilcoxon rank-sum tests for continuous measures and Pearson chi-square tests for categorical measures.

### Ethical Considerations

For individuals enrolled in the service component of this project who were also interested in participating in the research component, the research staff reviewed the informed consent document via phone or in person. Verbal or written consent was documented for all research participants. To uphold participant privacy and confidentiality, all engagement data was deidentified before analysis. Only members of the study team (eg, eCoaches) involved in participant enrollment and care provision had access to deidentified survey and messaging data. Through a contingency management component of this program, research participants could earn gift card incentives for completing the baseline survey (US $30) and specific activities in the app (eg, send your eCoach a message, review specific in-app course content) over the 1-month study period. All program participants (ie, research and nonresearch uMAT-R users) were informed of contingency management incentive structures at the time of enrollment. This study was approved by the Washington University Institutional Review Board (IRB 201805132, 201910161).

## Results

A total of 712 individuals were granted access to the uMAT-R application between January 1, 2020, and April 1, 2022, 702 of whom were recruited from general SUD recovery organizations (98.6%) and 10 of whom were recruited through the justice center (1%). A total of 17 individuals (2%) were excluded from the analysis because their uptake and engagement data were unavailable from iTether. Of the 695 included individuals, 446 (64.2%) logged into the uMAT-R application at least once during the 30 days following enrollment into the service project with a median number of logins of 2 (IQR 0-8; Table 1). The median number of logins among those who ever logged in was 5 (IQR 2-13; Table 1). Approximately half of those who had ever logged into the app (227/446; 50.9%) sent at least one eCoach message. The median number of messages sent among those who ever logged in was 1 (IQR 0-3; Table 1).

**Table 1:** Engagement in the uMAT-R app to support recovery from opioid and stimulant use disorders among individuals enrolled in the Missouri Opioid State Targeted Response Service Grant project between January 2020 and April 2022.

Engagement metric	All enrolled individuals (N=695)	Enrolled individuals with baseline survey data (n=498)	Enrolled individuals without baseline survey data (n=197)
At least one login, n (%)	446 (64.2)	379 (76.1)^a^	67 (34)^a^
Total logins, median (IQR) in total sample	2 (0-8)	4 (1-11)^a^	0 (0-1)^a^
Total logins, median (IQR) among those who logged in at least once	5 (2-13)	6 (2-16)^a^	1 (1-5)^a^
At least one eCoach message sent, n (% of those who logged in at least once)	227 (50.9)	211 (55.7)^a^	16 (24)^a^
Total eCoach messages sent, median (IQR) in total sample	0 (0-1)	0 (0-2)^a^	0 (0-0)^a^
Total eCoach messages sent, median (IQR) among those who logged in at least once	1 (0-3)	1 (0-4)^a^	0 (0-0)^a^

^a^These data indicate statistical significance at *P*<.001.

Baseline survey data completed within 7 days of enrollment into the service program was available for 498 of the 695 included individuals (71.7%). App uptake and engagement were greater among those who participated in the research component of the project compared to those who did not (Table 1). Of the 498 individuals with survey data, 379 (76.1%) logged into the uMAT-R app at least once during the 30 days following enrollment with a median number of logins of 4 (IQR 1-11; Table 1). The median number of logins among those who ever logged in was 6 (IQR 2-16; Table 1). Approximately 56% of those with survey data who had ever logged into the app (211/379) sent at least one message to their eCoach. The median number of messages sent was 1 (IQR 0-4) among research participants who ever logged in (Table 1).

Of the 498 individuals with baseline survey data, most were women (275/498; 55.2%), less than 50 years of age (435/491; 88.6%), White, non-Hispanic (382/493; 77.5%), unemployed (292/492; 59.3%), and stably housed (361/491; 73.5%; Table 2). Approximately 40% of the population was on Medicaid (211/492; 42.9%), 15% had private insurance (73/492; 15%), and just over half had any form of insurance coverage (275/491; 56%; Table 2).

**Table 2:** Login data from the uMAT-R application to support recovery from opioid and stimulant use disorders among 498 research participants enrolled in the Missouri Opioid State Targeted Response Service Grant between January 2020 and April 2022, overall and stratified by sociodemographic subgroup.

Sociodemographic characteristics				Total (N=498), n (%)				No logins (n=119), n (%)				At least one (n=379), n (%)				Total (n=497), median (IQR)			
**Gender^a^**																			
			Men			222 (44.6)				56 (25)				166 (74.8)				3 (0-8)^c^	
			Women			275 (55.2)				63 (23)				212 (77.1)				5 (1-14)^c^	
**Age (years)^b^**																			
			<50	436 (88.6)				98 (22)^c^				338 (77.5)^c^				4 (1-11)^c^			
			≥50	56 (11)				20 (36)^c^				36 (64)^c^				2 (0-6)^c^			
**Race and ethnicity^b^**																			
	Non-Hispanic White			382 (77.5)				82 (21)^c^				300 (78.5)^c^				4 (1-11)			
	Non-White or Hispanic			111 (22.5)				37 (33)^c^				74 (67)^c^				2 (0-11)			
**Educational attainment^b^**																			
	<High school diploma				97 (20)				37 (38)^c^				60 (62)^c^				3 (0-12)		
	≥High school diploma				396 (80.3)				82 (21)^c^				314 (79.3)^c^				4 (1-10)		
**Employment^b^**																			
	Unemployed			292 (59.3)				79 (27)				213 (73)				3 (0-10)^c^			
	Employed			200 (40.7)				40 (20)				160 (80)				5 (1-13)^c^			
**Housing^b^**																			
			Unstable	130 (26.5)				36 (28)				94 (72)				3 (0-11)			
			Stable	361 (73.5)				83 (23)				278 (77)				4 (1-11)			
**Medicaid insurance coverage^b^**																			
	No						281 (57.1)				59 (21)				222 (79)				4 (1-13)^c^
	Yes						211 (42.9)				60 (28)				151 (40.5)				3 (0-10)^c^
**Private insurance coverage^b^**																			
	No			419 (85.2)				106 (25.3)				313 (74.7)				4 (0-11)			
	Yes			73 (15)				12 (16)				61 (16)				3 (1-9)			
**Any insurance coverage^b^**																			
		No		216 (44)				49 (23)				167 (77.3)				4 (1-13)			
		Yes		275 (56)				69 (25)				206 (74.9)				3 (0-10)			

^a^Data suppression: one individual identified as gender nonconforming so engagement data were suppressed.

^b^Missing: age=5; race and ethnicity=5; educational attainment=5; employment=6; housing=7; Medicaid=6; private insurance=5; any insurance=6.

^c^These values indicate statistical significance at *P*<.05.

Overall, uptake (ie, ever vs never logging in) varied by age, race and ethnicity, and educational attainment. Engagement varied by gender, age, race and ethnicity, educational attainment, employment, and Medicaid coverage (Table 2). Specifically, those under 50 (77.5% vs 64.3% among those 50 and older), White, non-Hispanic individuals (78.5% vs 66.7% among non-White or Hispanic individuals), and those with a high school diploma or higher (79.3% vs 61.9% among those with less than a high school diploma) were more likely to log into the app at least once compared to their counterparts (Table 2). The median number of logins was higher among women (median 5, IQR 1-14) compared to men (median 3, IQR 0-8), those under the age of 50 (median 4, IQR 1-11) compared to those aged 50 years and older (median 2, IQR 0-6), employed individuals (median 5, IQR 1-13) compared to unemployed individuals (median 3, IQR 0-10), and those who were not on Medicaid (median 4, IQR 1-13) compared to those who were (median 3, IQR 0-10; Table 2). Among those who logged into the app at least once, those under the age of 50 were more likely to send at least one message to their eCoach (195/338; 57.7%) compared to those aged 50 years or older (13/36; 36.1%), as were those with lower educational attainment (41/60; 68.3%) compared to those with higher educational attainment (167/314; 53.2%; Table 3). No sociodemographic differences were observed in the median number of eCoach messages sent among those who logged into the app at least once.

**Table 3:** Use of the electronic health coach messaging feature in the uMAT-R app to support recovery from opioid and stimulant use disorders among 379 research participants enrolled in the Missouri Opioid State Targeted Response Service Grant project between January 2020 and April 2022 who logged into uMAT-R at least once during the 30 days following study enrollment, overall and stratified by sociodemographic subgroup.

Sociodemographic characteristic				Total (N=379), n (%)			No eCoach message sent (n=171), n (%)			At least one eCoach message sent (n=208), n (%)			Total (n=379), median (IQR)		
**Gender^a^**															
			Men	166 (43.8)			76 (46)			90 (54)			1 (0-3)		
			Women	212 (55.9)			91 (43)			121 (57.1)			1 (0-4)		
**Age (years)^b^**															
		<50		338 (90.4)			143 (42.3)^c^			195 (57.7)^c^			1 (0-4)		
		≥50		36 (10)			23 (64)^c^			13 (36)^c^			0 (0-2)		
**Race and ethnicity^b^**															
	White, non-Hispanic			300 (80.2)			138 (46)			162 (54)			1 (0-3)		
	Non-White or Hispanic			74 (20)			28 (38)			46 (62)			1 (0-4)		
**Educational attainment^b^**															
		<High school^d^ diploma		60 (16)			19 (32)^c^			41 (68)^c^			2 (0-4)		
		≥High school diploma		314 (84)			147 (46.8)^c^			167 (53.2)^c^			1 (0-3)		
**Employment^b^**															
	Unemployed			213 (57.1)			99 (46)			114 (53.5)			1 (0-4)		
	Employed			160 (42.9)			66 (41)			94 (59)			1 (0-3)		
**Housing^b^**															
	Unstable			94 (25)			41 (44)			53 (56)			1 (0-3)		
	Stable			278 (74.7)			125 (45)			153 (55)			1 (0-4)		
**Medicaid coverage^b^**															
	No					222 (58.5)			96 (43)			126 (56.8)			1 (0-4)
	Yes					151 (40.5)			70 (46)			81 (54)			1 (0-3)
**Private insurance coverage^b^**															
		No		313 (83.7)			132 (42.2)			181 (57.8)			1 (0-4)		
		Yes		61 (16)			34 (56)			27 (44)			0 (0-3)		
**Any insurance coverage^b^**															
	No				167 (44.8)			65 (39)			102 (61.1)			1 (0-4)	
	Yes				206 (55.2)			101 (49)			105 (51)			1 (0-3)	

^a^Data suppression: one individual identified as gender nonconforming so engagement data were suppressed.

^b^Missing: age=5; race and ethnicity=5; educational attainment=5; employment=6; housing=7; Medicaid=6; private insurance=5; any insurance=6.

^c^These values indicate statistical significance at *P*<.05.

^d^HS: high school.

## Discussion

### Principal Findings

We explored uptake and engagement in a mobile app to support recovery among individuals who misuse opioids and stimulants in the Greater St. Louis area. Overall, approximately two-thirds of individuals who obtained access to the app logged in at least once within 30 days of gaining access. Of those who logged in, around half sent at least one message to their eCoach through the mobile app messaging feature. Uptake and engagement with the app were higher among those who chose to participate in the research component of this service project, which offered monetary incentives for engagement, compared to those who did not. Variability in uptake or engagement was also observed with respect to gender, age, race and ethnicity, educational attainment, employment, and Medicaid, but not any insurance coverage.

### Comparison to Previous Work

A majority of individuals given access to uMAT-R (446/695, 64%) logged into the app at least once within 30 days of being granted access, and approximately half of those who logged in at least once sent at least one in-app message to their eCoach (227/446, 51%), demonstrating the short-term viability of this app as a resource to those in recovery for opioid and stimulant use disorders in the Greater St. Louis area. Broadly, uptake and engagement with mobile apps to support healthy behaviors, including SUD recovery, have been shown to vary widely [5,15,23-29]. For example, in a study in which nearly 2000 individuals with an SUD were offered access to a smartphone app to support substance misuse recovery, just 40% of participants downloaded the app, and just 18% engaged with the app [24]. In another study, patients who were given access to the Addiction-Comprehensive Health Enhancement Support System, a smartphone app designed to provide continuing care to patients with alcohol use disorders, used the application on 40% of the days during the 8-month intervention period [29]. On the other hand, in a smaller pilot study (n=25) of the HOPE application, a smartphone app designed to support medication for OUD treatment during COVID-19 lockdowns, 88% of participants used a provider messaging feature during the first month following enrollment into the study [30]. While messaging use fell to approximately 50% by month six, 76% were still engaging with other app features at this time point [30]. Acceptable levels of early engagement with uMAT-R suggest user habits should be explored longitudinally to assess how engagement patterns might decline or otherwise change over time.

Individuals who chose to participate in the research component of this project were more likely to log in to and engage with uMAT-R compared to those who did not participate. Studies exploring the variability in the app or digital intervention engagement between those who are and are not under observation (eg, those who are and are not enrolled in a research study) have also demonstrated variability [31]. For example, in a study exploring engagement with the See Me Smoke-Free mHealth app, research participants were equally as likely as nonresearch participants who installed the app to open the app and update smoking cessation dates but more likely to engage with audio features within the app [31]. While our findings are in line with a phenomenon originally coined the “Hawthorne effect,” which, broadly, posits that people under observation behave differently than those who are not under observation [32], these results may also be attributable to the fact that those willing to enroll in research may differ from those who are unwilling to do so (eg, historically marginalized groups who have been systematically oppressed or harmed by the medical or research community may be less likely to enroll in research and less likely to take up or engage with mHealth apps) [31-33]. Alternatively, incentives for app engagement may have increased uptake and use among those enrolled in the research component of this program, as has been documented in other work [33]. While incentives and related forms of contingency management are common in usability studies and were provided for this real-world substance use recovery support project through funding from the Missouri Department of Health, the long-term feasibility of incorporating incentives for app or intervention use or engagement in other real-world settings should be considered [34].

Among those with baseline survey data, uptake and the types and degree of engagement varied by sociodemographic subgroups, in line with findings from numerous other health promotion mobile app studies [35-37]. Specifically, we observed variation in uptake by age, race and ethnicity, and educational attainment, and variation in engagement by gender, age, race and ethnicity, educational attainment, employment, and Medicaid coverage specifically. Though some discrepancies in findings have been observed in the literature [35,36,38], a growing body of research supports our results that older individuals and men are less likely to engage with mHealth apps compared to their counterparts [35-37]. It is also well-documented in the literature that those with lower (versus higher) educational attainment are less likely to engage with mHealth apps [35-37]; while we saw a similar trend in the uptake of uMAT-R, the opposite trend was observed in eCoach messaging among those who logged in (ie, those with lower educational attainment were more likely to send at least one message to their eCoach compared to those with higher educational attainment). It is possible those with lower educational attainment had less access to resources and were, therefore, more inclined to seek support and resources from an eCoach compared to those with higher educational attainment who logged in, or that lifestyle differences between these groups influenced uptake and engagement in distinct ways. Qualitative data exploring why these differences emerged is needed. Racial and ethnic differences in app use have also been observed in the literature (though less commonly), with White, non-Hispanic individuals more likely to demonstrate sustained engagement in mHealth apps compared to others [35,39]. Differences in uptake of and engagement with mHealth apps by employment and insurance coverage have been less commonly explored and documented [35,40].

Ultimately, the variation in findings related to sociodemographic correlates of mHealth app uptake and engagement, coupled with other research related to mHealth app uptake and engagement over time [10,41,42] suggests that mobile apps can be tailored to increase acceptability, appropriateness, or usability of apps, or improve app engagement in a range of sociodemographic subgroups. For example, ecological momentary assessment was found to be feasible in older adults when it was designed to be responsive to the unique needs and preferences of that specific subgroup of individuals [42]. User co-design (eg, human-centered design methods and other methods for incorporating end-users in app development) [43-46] has shown promise as a method for improving engagement in substance-use recovery apps across a range of settings. The success of these methods has been well-documented [47-49] and should be used to further refine uMAT-R to increase sustained and comprehensive engagement in older individuals, men, racial and ethnic minorities, those with higher educational attainment, unemployed individuals, and those on Medicaid who either logged in less frequently or were less likely to use the eCoach messaging feature of uMAT-R. Potential alterations may include more comprehensive or geographically diverse resource information; participant-preferred or option-based rewards or incentives for sustained engagement; use of eCoaches with diverse specialties (eg, cognitive behavioral therapy, dialectical behavior therapy) or backgrounds (eg, racial and ethnic minorities, those with personal substance misuse recovery experience, men) who can be individually paired with participants based on participant preferences or needs; and personalized lessons or highlighted app features based on individual participant preferences or needs.

Alternative opioid and stimulant misuse recovery support approaches and interventions (eg, peer navigation) or additional support in downloading, logging into, or accessing specific features of a mHealth app may also be needed to support older individuals, non-White or Hispanic individuals, and those with lower educational attainment who were less likely to ever log in to uMAT-R compared to their counterparts. In qualitative work exploring the implementation of a substance use recovery support mobile phone app, Lord and colleagues identified several facilitators of the implementation and uptake of the app in 4 service settings. Facilitators included staff training promoting organizational awareness about the app and emphasizing app priority as a clinical care tool; client encouragement related to app testing and use; client expectation setting about app use; and use of peer coaches and recurrent client-centered messaging to promote engagement [50]. This suggests partnering more closely with staff at existing recruitment centers may improve uptake and engagement outcomes. A systematic review of barriers and facilitators to user engagement with digital mental health interventions also found that individuals were more likely to uptake or engage with these types of interventions if they were trained on how to use them or received automated or therapist-delivered reminders to use the intervention [51]. Such strategies could also be used to increase the uptake, use, and usability of uMAT-R as well.

### Limitations

This study has limitations worth nothing. First, sociodemographic data were missing for approximately 30% of individuals who were granted access to the uMAT-R application but chose not to participate in the research component of the project. Second, variability in-app engagement was observed with respect to participation in the research component with less engagement among those who did not participate, potentially due to incentives for engagement among those enrolled in the research component of the program. As a result, the generalizability of findings regarding sociodemographic differences in engagement is limited, with findings also suggesting incentives or contingency management may be a necessary component to ensure engagement in mHealth apps aimed to support opioid and stimulant misuse recovery efforts. Third, other metrics of mobile application engagement (eg, duration of engagement, content accessed) were not adequately captured by iTether, the technology company supporting the design and maintenance of the uMAT-R application during the observation period. Fourth, we only explored engagement during a relatively brief follow-up period (ie, 1 month following enrollment into the program) and engagement has been shown to decline over time, potentially indicating estimates reported here are a “best-case scenario.” Finally, end-user perspectives on other app features that could have influenced app uptake and engagement (eg, acceptability, appropriateness, usability) were not measured, nor were reasons for engagement in substance use treatment.

### Future Directions

Future studies exploring mobile app engagement should aim to capture diverse perspectives on overall app design, reasons for enrollment into recovery programs and uMAT-R specifically, and engagement metrics over an extended follow-up period (eg, one year) to establish a more holistic picture of what works for whom, when, and for how long.

### Conclusions

A majority of individuals granted access to the uMAT-R application logged in at least once in the first 30 days and of those who logged in, approximately half sent at least one in-app message to an eCoach. This suggests mobile apps, and uMAT-R in particular, may be a viable tool for supporting individuals in recovery from opioid and stimulant use disorders in Missouri. However, older individuals, racial and ethnic minorities, and those with lower educational attainment may be less likely to initiate the use of such interventions and may benefit from alternative mechanisms of substance use recovery support. Moreover, older individuals, men, racial and ethnic minorities, those with higher educational attainment, unemployed individuals, and those on Medicaid may be less likely to fully engage with such tools, or to engage over a sustained period and may benefit from app features tailored more specifically to their wants and needs. Incentives might also play an important role in increasing uptake and engagement with mHealth apps for improving SUD outcomes. Future studies should aim to explore how uptake and engagement in these types of tools vary over longer periods of time, how incentives influence app use, and the effect of engagement on recovery outcomes overall and in distinct socioeconomic subgroups.

## Abbreviations

eCoach: electronic health coach
OUD: opioid use disorders
SAMHSA: Substance Abuse and Mental Health Services Administration
SUD: substance use disorders

## Data Availability

The datasets generated or analyzed during this study are not publicly available due to the sensitive nature of this research but are available from the corresponding author on reasonable request.

## References

[1] Hawks L, Himmelstein DU, Woolhandler S, Bor DH, Gaffney A, McCormick D (2020). Trends in unmet need for physician and preventive services in the United States, 1998-2017. JAMA Intern Med.

[2] Messner EM, Probst T, O'Rourke T, Stoyanov S, Baumeister H (2019). mHealth Applications: Potentials, Limitations, Current Quality and Future Directions.

[3] Nilsen W, Kumar S, Shar A, Varoquiers C, Wiley T, Riley WT, Pavel M, Atienza AA (2012). Advancing the science of mHealth. J Health Commun.

[4] Nesvåg S, McKay JR (2018). Feasibility and effects of digital interventions to support people in recovery from substance use disorders: systematic review. J Med Internet Res.

[5] Neale J, Bowen AM (2022). Lessons for uptake and engagement of a smartphone app (SURE Recovery) for people in recovery from alcohol and other drug problems: interview study of app users. JMIR Hum Factors.

[6] Anderson-Lewis C, Darville G, Mercado RE, Howell S, Di Maggio S (2018). mHealth technology use and implications in historically underserved and minority populations in the United States: systematic literature review. JMIR mHealth uHealth.

[7] Friis-Healy EA, Nagy GA, Kollins SH (2021). It is time to REACT: opportunities for digital mental health apps to reduce mental health disparities in racially and ethnically minoritized groups. JMIR Ment Health.

[8] Bowen D, Jabson J, Kamen C (2016). mHealth: an avenue for promoting health among sexual and gender minority populations?. Mhealth.

[9] Johnston DC, Mathews WD, Maus A, Gustafson DH (2019). Using Smartphones to Improve Treatment Retention Among Impoverished Substance-Using Appalachian Women: A Naturalistic Study.

[10] Lunn MR, Capriotti MR, Flentje A, Bibbins-Domingo K, Pletcher MJ, Triano AJ, Sooksaman C, Frazier J, Obedin-Maliver J (2019). Using mobile technology to engage sexual and gender minorities in clinical research. PLoS One.

[11] Hales S, Turner-McGrievy GM, Wilcox S, Fahim A, Davis RE, Huhns M, Valafar H (2016). Social networks for improving healthy weight loss behaviors for overweight and obese adults: a randomized clinical trial of the social pounds off digitally (Social POD) mobile app. Int J Med Inform.

[12] Huberty J, Green J, Glissmann C, Larkey L, Puzia M, Lee C (2019). Efficacy of the mindfulness meditation mobile app 'Calm' to reduce stress among college students: randomized controlled trial. JMIR mHealth uHealth.

[13] Pallejà-Millán M, Rey-Reñones C, Barrera Uriarte ML, Granado-Font E, Basora J, Flores-Mateo G, Duch J (2020). Evaluation of the Tobbstop mobile app for smoking cessation: cluster randomized controlled clinical trial. JMIR Mhealth Uhealth.

[14] Leightley D, Williamson C, Rona RJ, Carr E, Shearer J, Davis JP, Simms A, Fear NT, Goodwin L, Murphy D (2022). Evaluating the efficacy of the Drinks:Ration mobile app to reduce alcohol consumption in a help-seeking military veteran population: randomized controlled trial. JMIR mHealth uHealth.

[15] Patterson Silver Wolf DA, Ramsey AT, Epstein J, Beeler-Stinn S, Deer AAB (2022). Bridges to sobriety: testing the feasibility and acceptability of a mobile app designed to supplement an adolescent substance use disorder treatment program. Clin Soc Work J.

[16] Hendrikoff L, Kambeitz-Ilankovic L, Pryss R, Senner F, Falkai P, Pogarell O, Hasan A, Peters H (2019). Prospective acceptance of distinct mobile mental health features in psychiatric patients and mental health professionals. J Psychiatr Res.

[17] Perski O, Blandford A, West R, Michie S (2017). Conceptualising engagement with digital behaviour change interventions: a systematic review using principles from critical interpretive synthesis. Transl Behav Med.

[18] Latulippe K, Hamel C, Giroux D (2017). Social health inequalities and eHealth: a literature review with qualitative synthesis of theoretical and empirical studies. J Med Internet Res.

[19] Ahmad F, Cisewski J, Rossen L, Sutton P (2022). Provisional drug overdose death counts. CDC.

[20] High PM, Marks K, Robbins V, Winograd R, Manocchio T, Clarke T, Wood C, Stringer M (2020). State targeted response to the opioid Crisis grants (opioid STR) program: preliminary findings from two case studies and the national cross-site evaluation. J Subst Abuse Treat.

[21] Cavazos-Rehg PA, Krauss MJ, Costello SJ, Ramsey AT, Petkas D, Gunderson S, Bierut LJ, Marsch LA (2020). Delivering information about medication assisted treatment to individuals who misuse opioids through a mobile app: a pilot study. J Public Health (Oxf).

[22] Decisions in recovery: treatment for opioid use disorders. SAMHSA Publications and Digital Products.

[23] Ubhi HK, Michie S, Kotz D, Wong WC, West R (2015). A mobile app to aid smoking cessation: preliminary evaluation of SmokeFree28. J Med Internet Res.

[24] Wilde JA, Zawislak K, Sawyer-Morris G, Hulsey J, Molfenter T, Taxman FS (2023). The adoption and sustainability of digital therapeutics in justice systems: a pilot feasibility study. Int J Drug Policy.

[25] Richterman A, Ghadimi F, Teitelman AM, Moore K, Acri T, North H, Lopez K, Ou V, Van Pelt AE, Momplaisir F (2023). Acceptability and feasibility of a mobile phone application to support HIV pre-exposure prophylaxis among women with opioid use disorder. AIDS Behav.

[26] Wang G, Wu B, Chen J, Yu G, Lin D, Wang G, Bai Z (2021). A novel mHealth App (RyPros) for prostate cancer management: an accessibility and acceptability study. Transl Androl Urol.

[27] Xiong X, Braun S, Stitzer M, Luderer H, Shafai G, Hare B, Stevenson M, Maricich Y (2023). Evaluation of real-world outcomes associated with use of a prescription digital therapeutic to treat substance use disorders. Am J Addict.

[28] Milne-Ives M, Lam C, De Cock C, Van Velthoven MH, Meinert E (2020). Mobile apps for health behavior change in physical activity, diet, drug and alcohol use, and mental health: systematic review. JMIR mHealth uHealth.

[29] Gustafson DH, McTavish FM, Chih M, Atwood AK, Johnson RA, Boyle MG, Levy MS, Driscoll H, Chisholm SM, Dillenburg L, Isham A, Shah D (2014). A smartphone application to support recovery from alcoholism: a randomized clinical trial. JAMA Psychiatry.

[30] Hodges J, Waselewski M, Harrington W, Franklin T, Schorling K, Huynh J, Tabackman A, Otero K, Ingersoll K, Tiouririne NA, Flickinger T, Dillingham R (2022). Six-month outcomes of the HOPE smartphone application designed to support treatment with medications for opioid use disorder and piloted during an early statewide COVID-19 lockdown. Addict Sci Clin Pract.

[31] Schmidt CA, Romine JK, Bell ML, Armin J, Gordon JS (2017). User participation and engagement with the See Me Smoke-Free mHealth app: prospective feasibility trial. JMIR mHealth uHealth.

[32] Sedgwick P, Greenwood N (2015). Understanding the Hawthorne effect. BMJ.

[33] Goyal S, Morita PP, Picton P, Seto E, Zbib A, Cafazzo JA (2016). JMIR mHealth uHealth.

[34] Nuijten R, Van Gorp P, Khanshan A, Le Blanc P, Kemperman A, van den Berg P, Simons M (2021). Health promotion through monetary incentives: evaluating the impact of different reinforcement schedules on engagement levels with a mHealth app. Electronics.

[35] Szinay D, Forbes CC, Busse H, DeSmet A, Smit ES, König LM (2023). Is the uptake, engagement, and effectiveness of exclusively mobile interventions for the promotion of weight-related behaviors equal for all? A systematic review. Obes Rev.

[36] Bol N, Helberger N, Weert JCM (2018). Differences in mobile health app use: a source of new digital inequalities?. Inf Soc.

[37] Carroll JK, Moorhead A, Bond R, LeBlanc WG, Petrella RJ, Fiscella K (2017). Who uses mobile phone health apps and does use matter? A secondary data analytics approach. J Med Internet Res.

[38] Nelson LA, Coston TD, Cherrington AL, Osborn CY (2016). Patterns of user engagement with mobile- and web-delivered self-care interventions for adults with T2DM: a review of the literature. Curr Diab Rep.

[39] Mitchell M, White L, Oh P, Alter D, Leahey T, Kwan M, Faulkner G (2017). Uptake of an incentive-based mHealth app: process evaluation of the carrot rewards app. JMIR mHealth uHealth.

[40] Demographics of mobile device ownership and adoption in the United States. Pew Research Center.

[41] Szinay D, Jones A, Chadborn T, Brown J, Naughton F (2020). Influences on the uptake of and engagement with health and well-being smartphone apps: systematic review. J Med Internet Res.

[42] Ramsey AT, Wetherell JL, Depp C, Dixon D, Lenze E (2016). Feasibility and acceptability of smartphone assessment in older adults with cognitive and emotional difficulties. J Technol Hum Serv.

[43] Bartholomew LK, Parcel GS, Kok G (1998). Intervention mapping: a process for developing theory- and evidence-based health education programs. Health Educ Behav.

[44] Bartholomew Eldrigde LK, Parcel GS, Kok G, Gottlieb NH, Schaalma H, Markham C, Tyrrell S, Shegog R, Fernández M, Mullen PD, Gonzales V, Tortolero-Luna G, Partida S (2016). Planning Health Promotion Programs: An Intervention Mapping Approach.

[45] Boy GA (2013). Orchestrating Human-Centered Design.

[46] Waselewski ME, Flickinger TE, Canan C, Harrington W, Franklin T, Otero KN, Huynh J, Waldman ALD, Hilgart M, Ingersoll K, Ait-Daoud Tiouririne N, Dillingham RA (2021). A mobile health app to support patients receiving medication-assisted treatment for opioid use disorder: development and feasibility study. JMIR Form Res.

[47] Saunders CH, Sierpe A, Stevens G, Elwyn G, Cantrell M, Engel J, Gonzalez M, Hayward M, Huebner J, Johnson L, Jimenez A, Little NR, McKenna C, Onteeru M, Oo Khine M, Pogue J, Salinas Vargas JL, Schmidt P, Thomeer R, Durand M, CONFIDENT Study Long-Term Care Partners (2022). Co-development of a web application (COVID-19 Social Site) for long-term care workers ('Something for Us'): user-centered design and participatory research study. J Med Internet Res.

[48] Hardy A, Wojdecka A, West J, Matthews E, Golby C, Ward T, Lopez ND, Freeman D, Waller H, Kuipers E, Bebbington P, Fowler D, Emsley R, Dunn G, Garety P (2018). How inclusive, user-centered design research can improve psychological therapies for psychosis: development of SlowMo. JMIR Ment Health.

[49] Hemingway C, Baja ES, Dalmacion GV, Medina PMB, Guevara EG, Sy TR, Dacombe R, Dormann C, Taegtmeyer M (2019). Development of a mobile game to influence behavior determinants of HIV service uptake among key populations in the Philippines: user-centered design process. JMIR Serious Games.

[50] Lord S, Moore SK, Ramsey A, Dinauer S, Johnson K (2016). Implementation of a substance use recovery support mobile phone app in community settings: qualitative study of clinician and staff perspectives of facilitators and barriers. JMIR Ment Health.

[51] Borghouts J, Eikey E, Mark G, De Leon C, Schueller SM, Schneider M, Stadnick N, Zheng K, Mukamel D, Sorkin DH (2021). Barriers to and facilitators of user engagement with digital mental health interventions: systematic review. J Med Internet Res.

